# Incidence trends and age-period-cohort analysis of Alzheimer’s disease and other dementias in the world and China from 1990 to 2021: analyses based on the Global Burden of Disease Study 2021

**DOI:** 10.3389/fneur.2025.1628577

**Published:** 2025-07-04

**Authors:** Xuewang Zhang, Hanxin Lu, Jun Xiong

**Affiliations:** ^1^School of Public Administration, Hangzhou Normal University, Hangzhou, China; ^2^Engineering Research Center of Mobile Health Management System, Ministry of Education, Hangzhou, China; ^3^Belgium-Zhejiang Joint Laboratory for Disorders of Consciousness, Hangzhou, China

**Keywords:** incidence trend, age-period-cohort analysis, ADOD, GBD, China

## Abstract

**Background:**

With the accelerating global population aging, the incidence rates of Alzheimer’s disease and other dementias (ADOD) continue to rise sharply, presenting a pressing public health challenge worldwide. In China, where population aging is occurring at an unprecedented rate, the epidemiological trajectory of ADOD has garnered significant attention. The marked increase in incidence rates has imposed a substantial burden on both society and families, driven by direct healthcare costs, long-term care demands, and productivity losses among caregivers.

**Methods:**

This study utilized the age-period-cohort model to analyze the changes in the incidence and disability-adjusted life years (DALYs) rate of ADOD among the elderly population in China from 1990 to 2021, considering the dimensions of age, period, and cohort. The necessary data for this analysis were sourced from the Global Burden of Disease (GBD) 2021.

**Results:**

From 1990 to 2021, the incidence, prevalence, and DALYs of ADOD have significantly risen both globally and in China, with the growth rates in China notably surpassing the global average. China’s age-standardized incidence rate demonstrated an annual percent change of 0.921 (95% UI: 0.817, 1.025), with females (1.107) exhibiting a higher trend compared to males (0.695). The Average Annual Percentage Change (AAPC) for prevalence reached 5.995 (95% UI: 5.327, 6.664), significantly exceeding the global figure of 0.617. Age-effect analysis indicated that the effect coefficient for the 60–64 age group was 197.30, escalating dramatically to 3698.13 for the 85–89 age group, thus highlighting an exponential increase in risk with advancing age. The period effect peaked at 1.04 during 2018–2021, which is associated with accelerated aging and improvements in diagnostic techniques. In cohort analysis, the risk for individuals born between 1950 and 1954 increased compared to those born between 1895 and 1899.

**Conclusion:**

China is confronted with a dual challenge: the escalating burden of ADOD and a rapidly aging population, with female populations disproportionately affected. Urgent, gender-sensitive interventions are essential to enhance early detection, improve long-term care, and address modifiable risk factors. This study emphasizes the necessity for sustained investments in public health infrastructure and research to mitigate the increasing societal and economic impacts of ADOD.

## 1 Introduction

Alzheimer’s disease and other dementias are among the leading public health concerns, and considered as one of the most pressing health issues in the world (1). There are over 50 million people worldwide living with dementia in 2020. This number will almost double every 20 years, reaching 82 million in 2030 and 152 million in 2050. Much of the increase will be in developing countries (2). According to the latest data released by the World Health Organization, the top 10 causes of death accounted for 39 million deaths, or 57% of the total 68 million deaths worldwide. And Alzheimer’s disease and other forms of dementia ranked as the seventh leading cause of death, killing 1.8 million lives (3). China has the largest number of dementia patients in the world, accounting for about one-fourth of dementia patients globally, bringing a heavy health and economic burden (4).

Despite extensive scientific inquiry, neurodegenerative diseases including Alzheimer’s disease and other dementias, remain uncurable, posing a growing challenge to public health systems and caregiving networks worldwide as populations continue to age (5). Alzheimer’s disease and other forms of dementia not only lead to irreversible cognitive decline in patients but also provoke behavioral abnormalities and a loss of independent living capabilities. This situation imposes a significant caregiving burden and economic strain on families, with direct healthcare costs constituting 3.2% of global healthcare expenditures (21). In China, approximately 15 million patients and their families confront long-term care requirements, with caregivers facing an annual average work-loss cost of 3.2 trillion Chinese yuan (22).

Despite existing research highlighting the global burden of Alzheimer’s disease and other dementias, significant research gaps persist in China concerning the epidemiological patterns and risk factors of these diseases. This study aims to address these gaps by analyzing trends in the incidence, prevalence, mortality, and DALYs of ADOD in China from 1990 to 2021, utilizing data from the Global Burden of Disease Study 2021 (GBD 2021). Furthermore, this study will employ age-period-cohort (APC) analysis to investigate the underlying driving factors contributing to these trends. The findings are anticipated to provide scientific evidence for developing targeted prevention and management strategies aimed at mitigating the burden of Alzheimer’s disease and other dementias in China.

## 2 Methods

### 2.1 Data source

The data required for this study on Alzheimer’s disease and other dementias were obtained from GBD 2021,and can also be accessed at https://ghdx.healthdata.org/gbd-2021. This comprehensive database provides global, regional, and national data on a wide array of diseases from 1980 to 2021, including detailed disease burden statistics for Alzheimer’s disease and other dementias in China. Additionally, the GBD database offers information on incidence, prevalence, mortality, years of life lost (YLL), years lived with disability (YLD), and DALY related to 369 diseases and injuries. The data of GBD mainly come from censuses, surveys, hospital records, and administrative records. It follows the Guidelines for Accurate and Transparent Health Estimates Reporting (GATHER) in a standardized and reproducible manner (6). GBD used case definitions from the Diagnostic and Statistical Manual of Mental Disorders (DSM; DSM-III, DSM-IV, or DSM V), which are used in surveys and cohort studies, as well as from the International Classification of Diseases (ICD; ICD-8, ICD-9, and ICD-10), which are used in vital registration and claims data sources (7). This study was approved by the Ethics Committee of Hangzhou Normal University, and the use of GBD (Global Burden of Disease) data adheres to the *Global Health Data Ethics Guidelines*.

### 2.2 Data analysis

In our data analysis, we comprehensively describe the trends in incidence, prevalence, deaths, and DALYs, as well as age-standardized rates (ASRs), disaggregated by gender, age, and year. We calculated age-standardized incidence rates (ASIRs), prevalence rates (ASPRs), and DALYs (8), incorporating 95% uncertainty intervals (UIs) to estimate the disease burden. These UIs account for both the variance in parameter estimation and the uncertainties associated with data collection and model selection (9). Given that the population primarily affected by this disease is the elderly, specifically those aged 60 and above, we only analyze the disease burden of ADOD among adults aged ≥60 years from 1990 to 2021 (10).

### 2.3 Research methods

#### 2.3.1 Time trend analysis

A time series analysis approach was employed, using years as the time axis to conduct an overall trend analysis of the disease burden of Alzheimer’s disease in China from 1990 to 2021. By plotting curves of disease burden indicators over time, the long-term trends of disease incidence were visually represented, including both upward and downward trends as well as fluctuations. Log-linear regression was employed to calculate the Average Annual Percentage Change (AAPC), which is utilized for assessing time trends in age-standardized rates. This method allowed for a deeper exploration of the underlying patterns of incidence trends and potential influencing factors, such as the comprehensive effects of socioeconomic development, advances in medical technology, and changes in population structure on these trends. We utilized Bayesian age-period-cohort models to predict the age-sex-specific number of new cases and deaths from 2022 to 2030 (11).

#### 2.3.2 Age-period-cohort analysis

This study employed an age-period-cohort (APC) model to analyze the changes in disease indicators for Alzheimer’s disease among the elderly population in China from 1990 to 2021, focusing on three dimensions: age, period, and cohort. The mathematical expression of the APC model is provided below: $\mu_{\mathrm{ik}}=\alpha_{i}+\beta_{j}+\gamma_{k}$ . This model was utilized to investigate the patterns of changes in incidence risk across these dimensions. To ensure the accuracy and reliability of the analysis results, professional statistical software was used for data processing and model construction. In this study, the R software package (version 4.2.3) and JD_GBDR (V2.37, Jingding Medical Technology Co., Ltd.) was used for the drawing of the figures.

## 3 Results

### 3.1 Population size and aging rate in China

From 1990 to 2021, the population of China aged 60 years and above exhibited a sustained growth trend, increasing from 91 million (8.5% of the total population) to 267 million (18.9%). This represents an average annual growth rate (AAGR) of 1.87%, which is significantly higher than the global average aging rate of 1.2% during the same period. Notably, the oldest-old population (aged 85 years and above) experienced particularly rapid growth, with its proportion surging from 0.5% (4.6 million) in 1990 to 1.8% (48.1 million) in 2021, reflecting an annual growth rate of 3.2%. This trend underscores the accelerated progression of population aging toward older age groups in China.

### 3.2 The disease trends from 1990 to 2021

At the global level, from 1990 to 2021, all indicators related to Alzheimer’s disease and other dementias—including the number of deaths, DALYs, prevalence, and incidence—exhibited significant upward trends. Females consistently demonstrated higher values than males across all indicators (deaths, DALYs, prevalence, and incidence), a discrepancy that may be attributed to females’ longer average life expectancy and distinct physiological characteristics. In China, the disease burden of ADOD also exhibited a significant upward trend across all indicators, with females again presenting higher values than males in each category. Compared to global trends, the growth rate of certain indicators in China surpassed the global average, despite baseline prevalence and incidence being lower than the global levels. Specific data are presented in Table 1.

**Table 1:** Deaths, DALYs, prevalence, and incidence of ADOD compared in 1990 and 2021.

Measure	Both sexes, number (95%UI)	Males, number (95%UI)	Males, number (95%UI)
Deaths, DALYs, Prevalence, and Incidence of ADOD in 1990			
Global			
Deaths	663294.44 (163580.41 to 1764991.32)	200382.91 (47756.90 to 550521.32)	462911.53 (115212.76 to 1215530.41)
DALYs	13572308.01 (6439340.75 to 29586865.34)	4465556.67 (2092517.14 to 9971767.69)	9106751.34 (4330350.39 to 19615097.65)
Prevalence	21799760.90 (19067087.20 to 24837693.05)	7656315.26 (6611275.56 to 8728099.19)	14143445.64 (12361842.01 to 16105291.71)
Incidence	3834525.86 (3367544.12 to 4358427.97)	1352318.94 (1177679.88 to 1551793.07)	2482206.92 (2183968.02 to 2820920.01)
China			
Deaths	119808.81 (28349.26 to 322103.28)	39596.64 (9246.58 to 113674.51)	80212.17 (19176.26 to 212442.40)
DALYs	2702484.11 (1239177.46 to 6085394.74)	972799.22 (434085.41 to 2307848.00)	1729684.89 (790231.83 to 3750293.66)
Prevalence	4024535.83 (3446397.50 to 4623086.05)	1511602.03 (1280687.86 to 1737519.77)	2512933.80 (2165,52.11 to 2892723.80)
Incidence	703178.00 (601505.73 to 808633.19)	260650.25 (222339.29 to 301788.28)	442527.74 (381566.69 to 507193.91)
Deaths, DALYs, Prevalence, and Incidence of ADOD in 2021			
Global			
Deaths	1952677.00 (512981.22 to 4984737.48)	626872.13 (153868.61 to 1677846.60)	1325804.87 (356478.48 to 3316,450.75)
DALYs	36332686.74 (17237624.04 to 76873276.22)	12524126.46 (5871758.06 to 27158682.55)	23808560.28 (11368142.66 to 49746523.96)
Prevalence	56856688.21 (49382064.01 to 64977511.92)	20753308.39 (17769420.13 to 23796797.51)	36103379.82 (31468184.99 to 41117469.75)
Incidence	9837055.84 (8620519.20 to 11163699.62)	3645491.67 (3144737.84 to 4183541.38)	6191564.17 (5432752.48 to 7009225.95)
China			
Deaths	491773.96 (124968.03 to 1330181.92)	163343.35 (40663.85 to 466659.82)	328430.61 (83714.81 to 862459.67)
DALYs	10072477.50 (4947154.11 to 22219153.71)	3572278.85 (1694715.53 to 8148477.56)	6500198.65 (3171764.56 to 13681028.96)
Prevalence	16990827.32 (14488494.04 to 19672741.19)	6162197.72 (5142286.06 to 7141800.17)	10828629.60 (9315735.20 to 12515957.37)
Incidence	2914112.02 (2504728.47 to 3350743.09)	1077297.18 (908448.48 to 1248194.17)	1836814.84 (1593651.47 to 2101342.51)

### 3.3 Average annual percentage change of the disease

In the research on Alzheimer’s disease and other dementias, the annual percent change (AAPC) of age-standardized incidence rates, prevalence rates, and DALYs are key indicators for assessing disease development trends and burden. Globally and within China, the incidence rate, prevalence rate, and DALYs rate of Alzheimer’s disease and other dementias exhibit upward trends. Gender disparities are evident across all indicators, with the increases in incidence rate, prevalence rate, and DALYs rate among women generally surpassing those observed in men. Notably, the AAPCs of age-standardized incidence rates, prevalence rates, and DALYs in China are significantly higher than global averages, indicating a rapid deterioration in the incidence, prevalence, and health burden of this disease within the country.

Focusing specifically on China, the AAPC of age-standardized incidence rates reached 0.921 (95% UI: 0.817, 1.025), which is substantially higher than the global level of 0.043 (95% UI: 0.021, 0.066). This stark contrast clearly indicates that the age-standardized incidence rates of Alzheimer’s disease and other dementias in China are rising at an alarming rate. Regarding gender differences, the AAPC of age-standardized incidence rates for women in China is 1.107 (95% UI: 0.988, 1.226), which is higher than that for men at 0.695 (95% UI: 0.627, 0.763). This disparity suggests that women experience a greater burden of this disease, potentially linked to their unique physiological structures and hormone levels. These physiological factors may render women more susceptible to Alzheimer’s disease and other dementias, particularly in the context of changes in age, period, and cohort.

The AAPC of age-standardized prevalence rates in China reaches a staggering 5.995 (95% UI: 5.327, 6.664), significantly surpassing the global rate of 0.617 (95% UI: 0.515, 0.719). In terms of the age-standardized DALYs rate, China’s AAPC is 0.653 (95% UI: 0.458, 0.847), indicating a significant increase. This reflects the continuous rise in the health burden associated with Alzheimer’s disease and other dementias in China. This trend aligns with the rapid escalation in both incidence and prevalence rates, suggesting that an increasing number of patients are experiencing a loss of health-related life years. Specific data are presented in Table 2.

**Table 2:** Percentage changes of age-standardized rates of all measures for ADOD in the world and China from 1990 to 2021.

Criteria	AAPC	
	China	Global
Age-standard incidence rate		
Both	0.92 (0.82,1.03)	0.04 (0.02,0.07)
Female	1.11 (0.99,1.23)	0.12 (0.10,0.14)
Male	0.70 (0.63,0.76)	0.08 (0.07,0.09)
Age-standard prevalence rate		
Both	6.00 (5.33,6.66)	0.62 (0.52,0.72)
Female	7.29 (6.50,8.08)	0.96 (0.84,1.07)
Male	4.74 (4.21,5.26)	0.47 (0.39,0.56)
Age-standard DALYs rate		
Both	0.65 (0.46,0.85)	0.14 (0.09,0.19)
Female	0.96 (0.68,1.24)	0.27 (0.21,0.33)
Male	0.99 (0.81,1.17)	0.23 (0.19,0.27)

### 3.4 Age-period-cohort analysis of the disease

Age exhibited a highly significant positive correlation with the risk of Alzheimer’s disease. A detailed data analysis revealed that the effect coefficient for the 60–64 age group was 197.30 (95% CI: 193.55, 201.12), indicating that the incidence risk at this age was relatively stable and low, although a baseline risk of disease presence was evident. As age increased, the incidence risk rose sharply: the effect coefficient surged to 3698.13 (95% CI: 3650.44, 3746.43) for the 85–89 age group, representing a substantial increase compared to the 60–64 age group. The age-effect curve illustrates a clear upward trend in the incidence rate (per 100,000 person-years) as age increases from 65 to 95 years. The incidence rate remains relatively low at 65 years, then rises rapidly, reaching a high level by 95 years. This confirms that age is a critical risk factor for the disease, with advancing age significantly increasing the risk of incidence (Table 3).

**Table 3:** Age-period-cohort model results of ADOD incidence in China from 1990 to 2021.

Factor	Effect coefficient	Upper	Lower
Age (years)			
60–64	197.30	201.12	193.55
65–69	342.36	347.66	337.14
70–74	679.01	687.94	670.19
75–79	1480.95	1498.10	1464.01
80–84	2637.79	2667.85	2608.08
85–89	3698.13	3746.43	3650.44
Period (years)			
1993–1997	0.96	0.98	0.95
1998–2002	0.98	1.00	0.97
2003–2007	1.00	1.00	1.00
2008–2012	0.99	1.00	0.97
2013–2017	1.03	1.05	1.02
2018–2021	1.04	1.06	1.03
Cohort (birth year)			
1895–1899	0.93	1.14	0.76
1900–1904	0.94	1.00	0.88
1905–1909	0.96	0.99	0.93
1910–1914	0.98	1.00	0.96
1915–1919	0.99	1.00	0.97
1920–1924	0.99	1.00	0.97
1925–1929	1.00	1.00	1.00
1930–1934	1.01	1.03	1.00
1935–1939	1.05	1.06	1.03
1940–1944	1.08	1.09	1.06
1945–1949	1.09	1.11	1.07
1950–1954	1.11	1.14	1.09
1955–1959	1.10	1.14	1.07

The period effect demonstrated distinct fluctuating patterns in the incidence trend of Alzheimer’s disease. During 1993–1997, the effect coefficient was 0.96 (95% CI: 0.95, 0.98), indicating a relatively stable and low incidence risk. However, from 2018 to 2021, the effect coefficient increased to 1.04 (95% CI: 1.03, 1.06), reflecting a notable upward trend in incidence risk. Between 1995 and 2020, the incidence rate ratio fluctuated, rising in some periods and declining in others, reflecting the comprehensive influence of social, economic, and environmental factors on disease risk across different eras. For example, periods of rapid economic development and significant lifestyle changes—characterized by increased stress, reduced physical activity, and dietary shifts—may have elevated incidence risk, while improvements in healthcare access and disease prevention awareness may have mitigated risk in other periods. Additionally, the implementation of public health policies and changes in environmental quality also impacted incidence rates.

**Figure 1:**
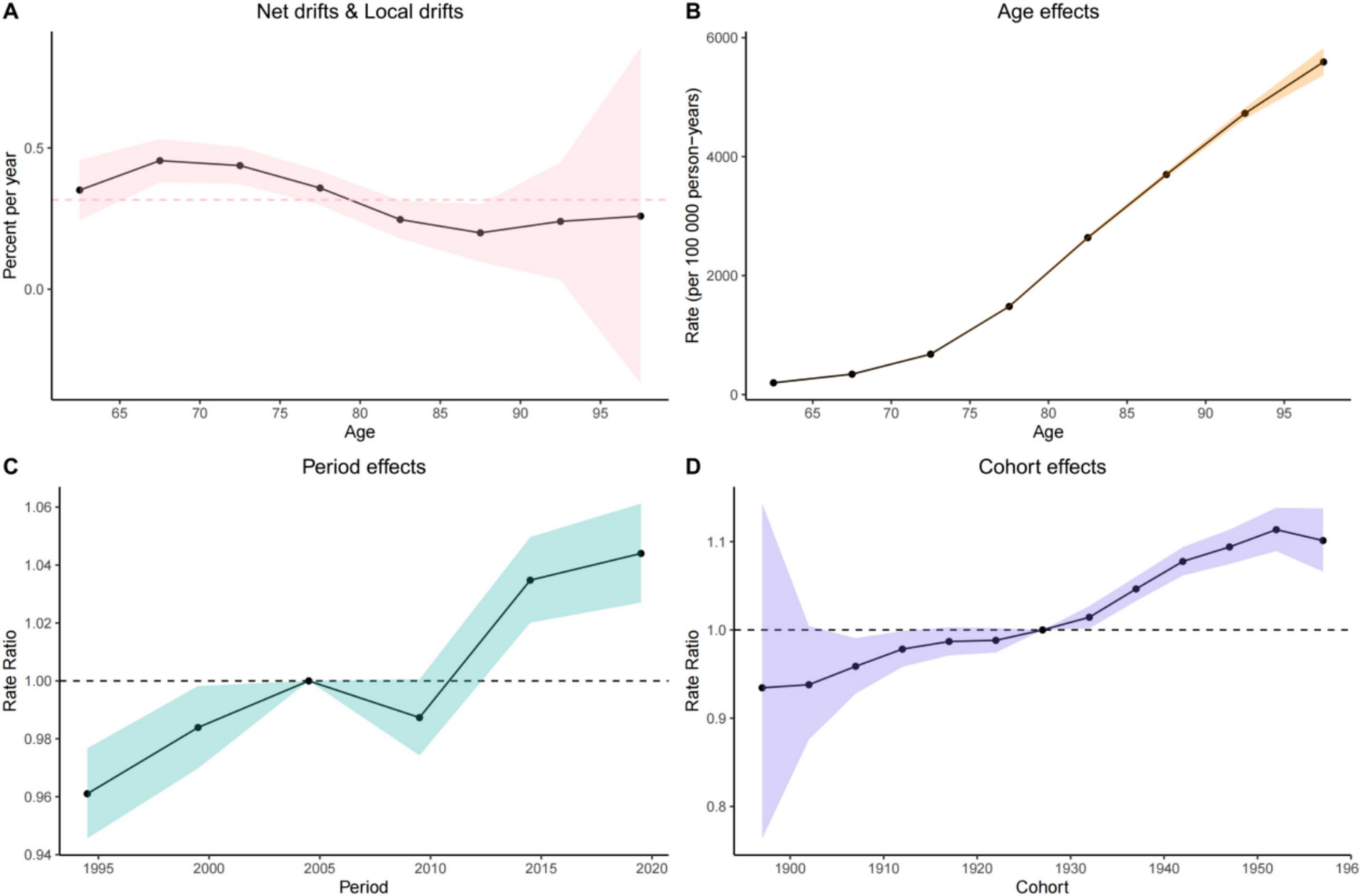
Age-period-cohort effects on incidence of ADOD in China from 1990 to 2021. **(A)** Quantification of relative changes in disease risk using Rate Ratio, used for comparing disease risks across different cohorts or periods. **(B)** Age effect analysis, showing the disease incidence rate in different age groups (per 100,000 person-years). **(C)** Period effect analysis, presenting the trend of disease rate ratio changes across different periods. **(D)** Cohort effect analysis, displaying the differences in disease rate ratios among different birth cohorts.

Cohort effect analysis revealed significant differences in the risk of Alzheimer’s disease across various birth cohorts. The birth cohort from 1895 to 1899 exhibited an effect coefficient of 0.93 (95% CI: 0.76, 1.14), indicating a relatively low incidence risk. In contrast, the cohort born between 1950 and 1954 showed an increase in this coefficient to 1.11 (95% CI: 1.09, 1.14), reflecting a marked rise in risk. The cohort trend curve illustrated dynamic changes in incidence risk for individuals born between 1900 and 1960: early cohorts (e.g., those born from 1900 to 1910) experienced relatively high risk, followed by a decline as birth years progressed, and a subsequent moderate increase in later cohorts. These patterns may be attributed to variations in living environments, nutritional status, and medical conditions encountered during each cohort’s formative years. Early cohorts faced greater life hardships and inferior healthcare, which weakened physical resilience and heightened risk. Conversely, later cohorts benefited from improved living standards but were also exposed to modern lifestyle risks such as chronic stress and unhealthy habits, potentially contributing to the subsequent rise in incidence risk (Figure 1).

### 3.5 Decomposition analysis of the disease

Incidence refers to the number of new cases occurring within a specific population over a defined period. The analysis indicates that the incidence in China is increasing, which can be attributed to several factors. A significant factor is population aging, as the likelihood of developing the disease escalates with age. Furthermore, lifestyle changes—such as decreased physical activity, poor dietary habits, and heightened stress levels in contemporary society—may also contribute to the rising incidence. Globally, similar trends are observed; however, the rate of increase may differ by region due to variations in economic development, healthcare quality, and population characteristics.

Prevalence denotes the total number of cases (both new and existing) within a population at a specific point in time. The decomposition of prevalence data reveals a marked increase both in China and globally. In China, the combination of rising incidence and enhanced survival rates—attributable to improved medical care—has resulted in a significant increase in prevalence. This rise not only reflects the current disease burden but also indicates a heightened demand for long-term care and medical resources. Additionally, gender differences are notable, as females generally exhibit a higher prevalence, which aligns with mortality and DALYs data.

The number of deaths resulting from the disease serves as a crucial indicator of its impact. By analyzing the death data, we observe distinct gender differences both globally and within China. Females generally exhibit a higher mortality rate associated with the disease, which may be linked to biological factors such as hormonal fluctuations and a longer life expectancy, resulting in prolonged exposure to the disease. Regionally, the death toll in China is influenced by its large population and the specific epidemiological characteristics of the disease. Analyzing age-specific mortality data allows us to comprehend the vulnerability of various age groups; for instance, the elderly population demonstrates a higher death rate, indicating an urgent need for targeted prevention and treatment strategies for this demographic.

DALYs combine years of life lost due to premature death with years lived with disability, offering a comprehensive assessment of disease burden. Analyzing DALYs data reveals the influence of age, gender, and region on the burden of disease. In China, the trends in DALYs associated with the disease differ from global patterns. Given the rapid aging of the population in China, DALYs may significantly increase in the future if effective intervention measures are not implemented, as the elderly are more susceptible to the disease, and a larger elderly population correlates with a heightened risk of disease-related disability and premature death. Globally, population growth and aging also contribute to an overall upward trend in DALYs.

**Figure 2:**
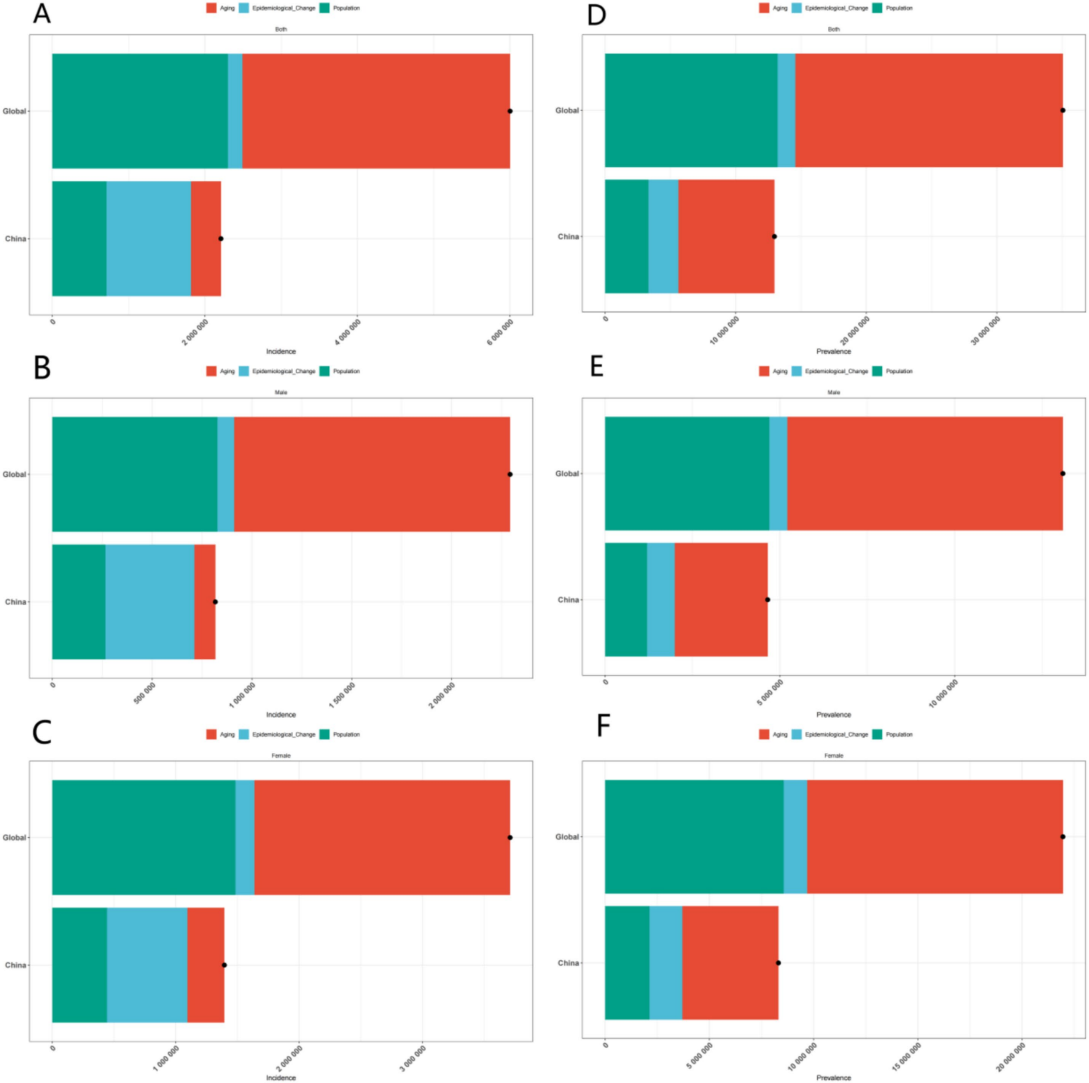
Gender-specific decomposition analysis of ADOD incidence and prevalence. **(A)** Decomposition analysis of ADOD incidence across all genders in China and globally. **(B)** Decomposition analysis of ADOD incidence across among males in China and globally. **(C)** Decomposition analysis of ADOD incidence across among females in China and globally. **(D)** Decomposition analysis of ADOD prevalence across all genders in China and globally. **(E)** Decomposition analysis of ADOD prevalence across among males in China and globally. **(F)** Decomposition analysis of ADOD prevalence across among females in China and globally.

**Figure 3:**
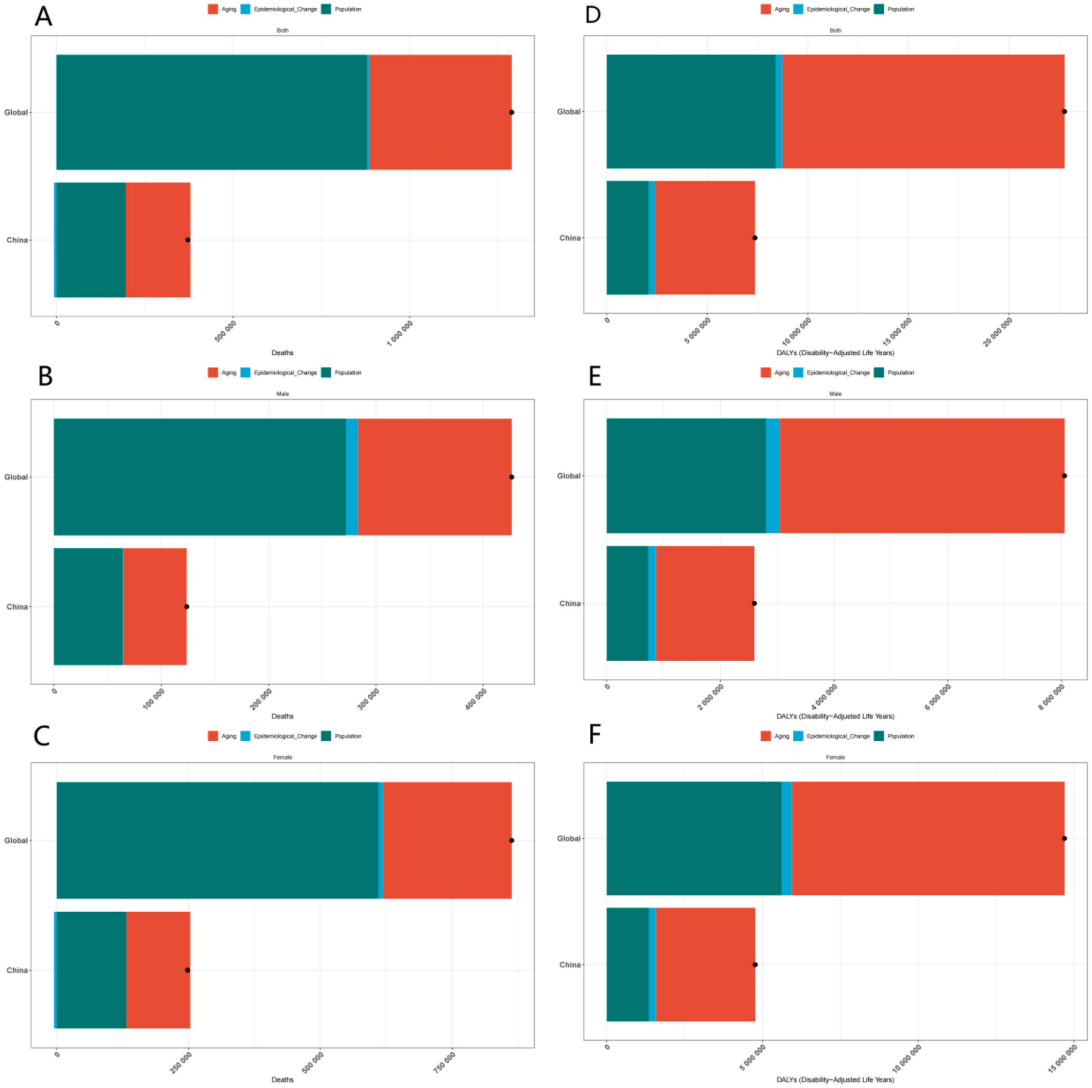
Gender-specific decomposition analysis of ADOD deaths and DALYs. **(A)** Decomposition analysis of ADOD deaths across all genders in China and globally. **(B)** Decomposition analysis of ADOD deaths across among males in China and globally. **(C)** Decomposition analysis of ADOD deaths across among females in China and globally. **(D)** Decomposition analysis of ADOD DALYs across all genders in China and globally. **(E)** Decomposition analysis of ADOD DALYs across among males in China and globally. **(F)** Decomposition analysis of ADOD DALYs across among females in China and globally.

In conclusion, the decomposition analysis of incidence, prevalence, deaths, and DALYs data elucidates the complex influences of age, gender, and region on the disease. This analysis provides a scientific foundation for developing targeted prevention and control strategies. Future research should concentrate on further investigating the underlying mechanisms of these disparities and on devising more effective intervention measures to mitigate the disease burden (Figures 2, 3).

### 3.6 Future predictions of the disease

The datasets featuring age-standardized rates per 100,000 from 1990 to 2030, with data post-2021 being projections, provide profound insights into the potential future trajectories of the disease.

The incidence data, represented as the age-standardized rate per 100,000 from 1990 to 2030, serves as a critical indicator for forecasting the disease’s future spread. In the post-2021 prediction period, an upward trend in the incidence rate would suggest a surge in new cases, potentially attributed to various factors such as environmental changes, demographic shifts (notably the aging population with increased susceptibility), and lifestyle modifications. An increasing incidence rate would place considerable strain on public health systems, necessitating additional resources for early detection, diagnosis, and treatment. Conversely, a downward trend would indicate the effectiveness of preventive measures, including vaccination campaigns, health education programs, and improvements in living conditions.

The prevalence data, illustrated as the age-standardized rate per 100,000 from 1990 to 2030, reflects the overall disease burden within the population at a specific time. For the years following 2021, a rising prevalence rate forecasts an increasing number of individuals living with the disease. This may result from a combination of heightened incidence and improved survival rates due to advancements in medical care. A continuously rising prevalence would impose an even greater burden on the healthcare system, social support networks, and families, heightening the demand for long-term care, rehabilitation services, and medical research aimed at developing more effective treatment modalities. Conversely, a declining prevalence rate would signify a positive outcome, indicating successful disease control through a comprehensive approach that encompasses prevention, treatment, and management strategies.

The age-standardized death rate per 100,000 exhibits fluctuations from 1990 to 2030. In the prediction period following 2021, a declining death rate may indicate advancements in medical treatment, improved disease management, and heightened public health awareness. For example, the development of more effective medications, the implementation of advanced early-detection screening programs, and public health initiatives that promote healthy lifestyles could contribute to this decline. Conversely, an increasing death rate may arise from emerging risk factors, including environmental changes, detrimental population-wide health behaviors such as a rise in smoking or sedentary lifestyles, and the ineffectiveness of existing treatment methods against new disease variants.

The DALYs data demonstrates an evolving trend over time. The overall direction indicated on the graph suggests a significant shift in the disease-related burden measured in DALYs. If the current trend continues beyond 2021, it is likely that the DALYs rate will increase, primarily due to the ongoing aging of the population. Elderly individuals are more susceptible to diseases and often experience higher rates of associated disabilities. Without substantial medical breakthroughs or effective preventive interventions, this upward trend may intensify. An increasing DALYs rate would not only diminish the quality of life for patients but also impose a greater burden on society, escalating the demand for healthcare resources and long-term care services.

**Figure 4:**
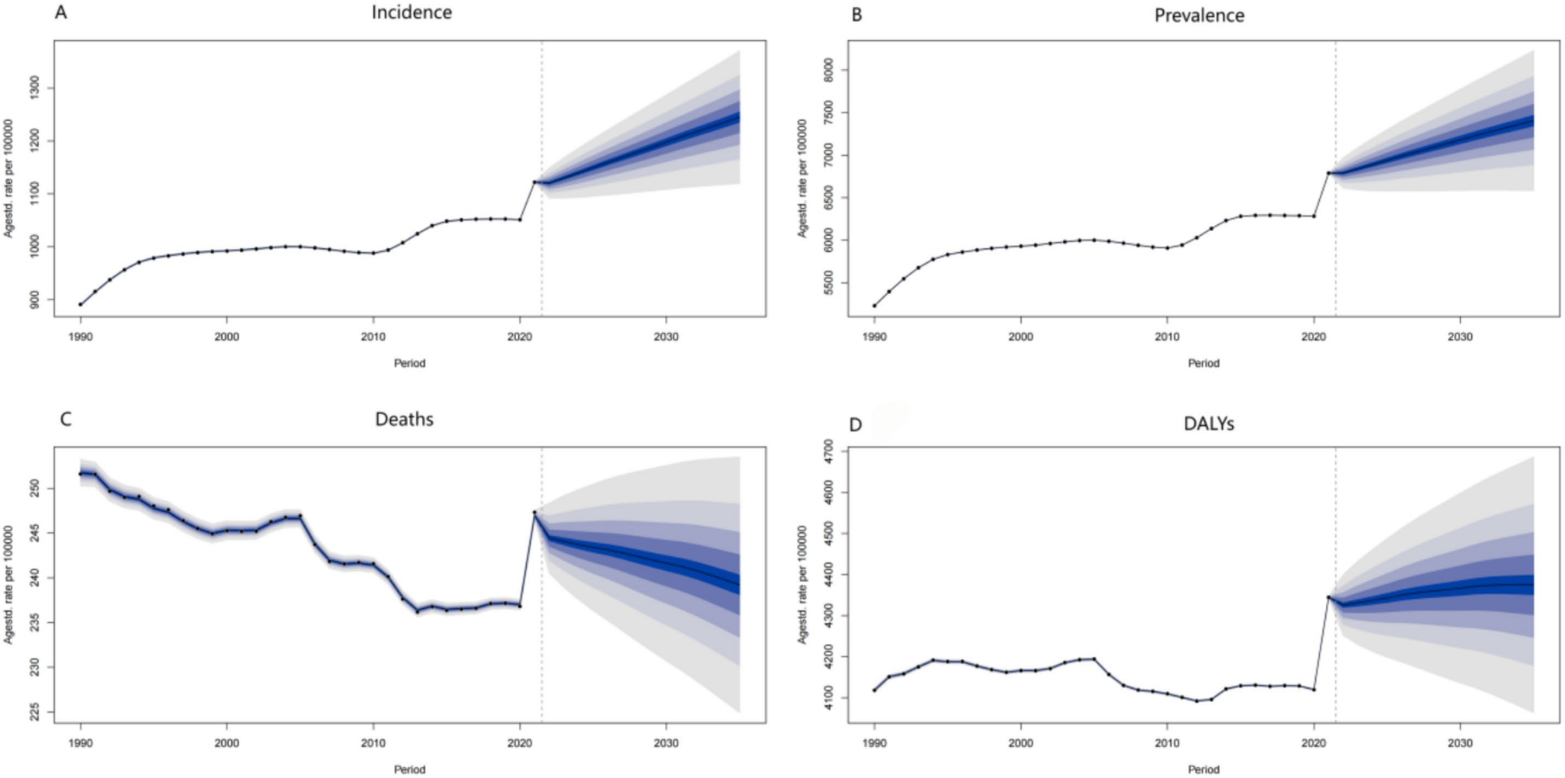
ADOD’s future predictions in Incidence, Prevalence, Deaths, and DALYs. **(A)** Future predictions of ADOD incidence rate to 2030. **(B)** Future predictions of ADOD prevalence rate to 2030. **(C)** Future predictions of ADOD deaths rate to 2030. **(D)** Future predictions of ADOD DALYs rate to 2030.

In summary, these future projections based on the provided data highlight the importance of continuous surveillance of disease trends. Proactive strategies for disease prevention, early detection, and improved treatment are crucial. By implementing such measures, we can potentially mitigate the adverse impacts of diseases on individuals and society, and strive to reduce the overall disease burden in the future (Figure 4).

## 4 Discussion

### 4.1 Key findings and global context

The present study provides a comprehensive analysis of the epidemiological trends of Alzheimer’s disease and other dementias in China from 1990 to 2021, leveraging data from the Global Burden of Disease Study 2021. The most striking finding is the rapid escalation of disease burden in China, with all indicators—incidence, prevalence, deaths, and DALYs—showing pronounced upward trends, often outpacing global averages. For instance, the number of deaths in China nearly quadrupled from 1990 to 2021, compared to a near tripling globally (12), highlighting the urgent need for targeted interventions in the context of China’s rapidly aging population. In the Asian region, the growth rates of deaths due to ADOD and DALYs reached 297.34 and 249.54%, respectively. As a country with a rapidly increasing aging population—where the aging rate rose from 8.5% in 1990 to 18.9% in 2021—China’s burden of ADOD, alongside that of Japan (whose aging rate increased from 12 to 28%), collectively exemplifies the ‘high-growth-rate’ characteristic of East Asia (13). Gender disparities were consistent across all metrics, with females consistently bearing a heavier burden, likely influenced by longer life expectancy and biological factors that increase susceptibility to dementias (8).

### 4.2 Impact of modifiable risk factors

The prevalence of Alzheimer’s disease and other dementias is closely associated with multiple modifiable risk factors, which have become particularly prominent during China’s rapid urbanization. Physical inactivity represents a significant risk factor; the proportion of Chinese adults engaging in regular physical exercise declined from 60% in 1990 to 35% in 2021 (14), paralleling the global trend of increased dementia risk. Regarding dietary pattern changes, the rising consumption of processed foods has contributed to an increase in obesity prevalence, which reached 18% among individuals aged ≥60 years in 2021 and is significantly correlated with *β*-amyloid deposition (15). Additionally, annual average PM2.5 concentrations in some regions of China reach 50 μg/m^3^, which is 2.5 times higher than the World Health Organization standard. Long-term exposure to such pollution may accelerate cognitive decline by inducing neuroinflammation and oxidative stress (16). Notably, females exhibit greater sensitivity to air pollution due to fluctuations in estrogen levels, which may explain the gender disparity in age-accelerated population change (AAPC) rates (1.107 vs. 0.695) (17). Future longitudinal studies are warranted to quantify the attributable risks of these factors, thereby providing precise intervention targets for policies such as “Healthy China 2030.”

### 4.3 Biological and sociological explanations for gender differences

Women experience a higher disease burden in Alzheimer’s disease and other dementias due to complex biological mechanisms and social structural factors. Biologically, women exhibit greater sensitivity to the neurotoxicity associated with metabolic abnormalities. Notably, 76.8% of the DALYs attributed to high body mass index (BMI) in ADOD occur in women. In Asian women, each 1 kg/m^2^ increase in BMI correlates with a 7.2% increase in the risk of developing Alzheimer’s disease. This phenomenon is linked to the pronounced release of chronic inflammatory cytokines, such as IL-6 and TNF-*α*, which is influenced by the distribution of adipose tissue in women, primarily as subcutaneous fat (6, 18). In China, women with fasting blood glucose levels equal to or exceeding 7.0 mmol/L face a 41% higher risk of ADOD compared to men, likely due to the increased sensitivity to insulin resistance associated with estrogen deficiency (11). Sociologically, there are gender-differentiated exposures to smoking and alcohol consumption. The mortality rate from ADOD attributed to smoking is 3.8 times higher in men than in women, and men represent 63% of early-onset dementia cases (aged under 65 years). Additionally, globally, the rate of alcohol consumption among men is 2.4 times that of women. Alcohol-related brain injury in men more frequently presents as mixed dementia (vascular and Alzheimer’s pathology), whereas alcohol exposure in women is more likely to result in hippocampal atrophy (14).

### 4.4 Policy implications

Grassroots interventions can significantly enhance the early detection rate of cognitive impairments (6). It is recommended to implement an annual Mini-Mental State Examination (MMSE) for diabetic patients in primary healthcare institutions, such as community health service centers. Furthermore, gender-specific prevention and control strategies are essential. For women, it is crucial to address the increased metabolic risks associated with postmenopause by conducting routine blood glucose monitoring and cognitive assessments in gynecological menopause clinics. For men, the focus should be on mitigating risks related to smoking and excessive alcohol consumption (9).

### 4.5 Public health implications

The findings highlight China’s unique challenge: a dual burden of increasing incidence/prevalence and a rapidly aging society (8). The quadrupling of deaths and DALYs underscores the strain on healthcare resources and informal care networks. Given that females exhibit higher burdens across all indicators, gender-sensitive policies—such as targeted screening and mental health support—are imperative. The APC analysis also suggests that younger cohorts may face lower early-life risks, possibly due to improved education and living standards; however, the aging of these cohorts will still drive overall burden growth due to their sheer population size. In clinical practice, populations at risk for Alzheimer’s disease and other dementias are encouraged to take active control in various ways. These populations should develop self-management skills, which should be reinforced in clinical guidelines (19). Additionally, suitable training is required for both doctors and caregivers (13).

### 4.6 Limitations and future directions

Given that the GBD study relies on information from diagnosed cases, it is highly likely that the disease burden is underestimated. Therefore, this factor should be taken into account when interpreting the results (6). And GBD data relies on aggregate national-level estimates, potentially masking regional disparities within China (20). A subnational analysis that integrates socioeconomic factors, such as urban–rural differences and access to healthcare, could enhance these insights. For example, the dementia diagnosis rate in rural areas is only 50% of that in urban areas, potentially leading to an underestimation of the disease burden. Furthermore, the aging rate in eastern coastal areas (22%) surpasses that of western regions (16%). Additionally, the unequal distribution of medical resources—illustrated by the number of tertiary hospital beds per 1,000 population, which stands at 5.2 in the east compared to 3.1 in the west—may exacerbate regional disparities in disease burden. Future research should integrate provincial-level data and incorporate indicators of urban–rural educational levels (illiteracy rate: 12% in rural areas versus 4% in urban areas) and medical accessibility to further analyze the driving mechanisms behind these regional inequalities. Furthermore, the role of modifiable risk factors—including air pollution, diet, and physical activity—has not been thoroughly examined within the current framework, even though they are well-established contributors to dementia risk. Future research should prioritize longitudinal cohort studies to elucidate the intricate interactions among aging, environmental exposures, and genetic predispositions.

## 5 Conclusion

The study highlights China’s dual challenge of an increasing disease burden and demographic aging, necessitating the implementation of targeted strategies to enhance early detection, strengthen long-term care systems, and address modifiable risk factors. Gender-sensitive policies and investments in research for prevention and treatment are imperative to alleviate the strain on healthcare and social support networks. While limitations include aggregated national data and unexamined regional/socioeconomic disparities, this research provides a foundational framework for future studies to explore subnational trends and mechanistic drivers of ADOD (8).

The escalating burden of Alzheimer’s disease and other dementias in China necessitates urgent, evidence-based interventions to mitigate their impact on individuals, families, and society. Sustained efforts in public health, healthcare infrastructure, and research are essential for addressing the challenges posed by this rapidly evolving epidemic.

## Data Availability

The datasets presented in this study can be found in online repositories. The names of the repository/repositories and accession number(s) can be found at: https://ghdx.healthdata.org/gbd-2021.
